# Global Trends in Virtual Reality Research on Motor Rehabilitation from 2005 to 2025: A Bibliometric Analysis

**DOI:** 10.3390/healthcare14131976

**Published:** 2026-07-02

**Authors:** Yarong Kong, Ziyi Shu, Yoon-soo Han

**Affiliations:** Department of Physical Education, Kyungpook National University, Daegu 41566, Republic of Korea; kongyarong93@knu.ac.kr (Y.K.); szy15641885500@gmail.com (Z.S.)

**Keywords:** virtual reality, motor rehabilitation, research trend, bibliometrics, visualization analysis

## Abstract

**Highlights:**

**What are the main findings?**

- First, research on VR for motor rehabilitation grew markedly from 2005 to 2025, with rapid expansion after 2020.
- Second, the United States was the leading contributor, followed by Italy, China, and Canada, while McGill University was the most productive institution.
- Third, major research hotspots included post-stroke upper-limb recovery, gait and balance training, immersive VR, robot-assisted rehabilitation, and telerehabilitation.

**What are the implications of the main findings?**

- First, VR research has gradually become more closely connected with health-related fields, including rehabilitation, neurosurgery, and other clinical applications. In this context, the rapid increase in publications on VR-based motor rehabilitation after 2020 may be associated with several broader developments, such as advances in VR hardware, increasing clinical interest in digital rehabilitation, and the growing demand for remote or home-based rehabilitation during and after the COVID-19 pandemic.
- Second, the leading role of countries such as the United States, Italy, China, and Canada indicates that international collaboration is important for advancing this field, particularly because VR-based rehabilitation requires expertise from rehabilitation medicine, neuroscience, engineering, computer science, and human–computer interaction.
- Third, the concentration of research on stroke rehabilitation, gait and balance training, immersive VR, robot-assisted rehabilitation, and telerehabilitation suggests that future studies could place greater emphasis on specific patient groups, rehabilitation stages, intervention mechanisms, and implementation contexts. This would help move the field from broad trend identification toward more precise evidence for how VR-based interventions can be designed and applied in motor rehabilitation.

**Abstract:**

**Background:** Virtual reality (VR) has been increasingly used in motor rehabilitation over the past two decades, but the overall research landscape of this field has not been fully mapped from a bibliometric perspective. **Objective:** This study aimed to conduct a bibliometric analysis to determine the development of research on VR for motor rehabilitation, focusing on its knowledge structure, major research topics, and temporal changes in the field. **Methods:** A topic-based search combining VR- and motor rehabilitation-related terms was conducted in the Web of Science Core Collection for the period from 2005 to 2025, yielding 1232 publications. VOSviewer, CiteSpace, R, and Scimago Graphica were used to analyze publication trends, country and institutional contributions, author collaboration, journal and reference co-citation, keyword co-occurrence, citation bursts, and thematic evolution. **Results:** Publications increased in three stages: slow exploration, steady growth, and rapid expansion. The United States, Italy, China, and Canada were the leading contributors, with McGill University as the most productive institution. Research hotspots included gait and neurological rehabilitation, post-stroke upper-limb recovery, robotics- and neuroscience-integrated rehabilitation, and the rise of immersive VR technology. **Conclusions:** This study provides a bibliometric overview of research progress in the application of virtual reality technology to motor rehabilitation, offering systematic insights into the field’s knowledge structure, core research themes, evolutionary trajectory, and future research directions.

## 1. Introduction

Motor function provides an essential foundation for carrying out basic activities of daily living, so when it is impaired, individuals may experience varying degrees of restriction in mobility and everyday tasks, which can diminish their quality of life. In clinical practice, conditions such as stroke [1], Parkinson’s disease [2], and cerebral palsy [3] are commonly associated with upper- or lower-limb motor impairments [4], often accompanied by balance and gait abnormalities [5]. These difficulties can substantially affect patients’ overall well-being and independence in daily life. Therefore, alleviating motor dysfunction and promoting motor recovery have become central goals of rehabilitation medicine [6].

In traditional motor rehabilitation, face-to-face exercise guidance delivered by physical therapists remains a common approach, but this intervention places substantial demands on institutions and healthcare professionals while also being costly and inconvenient for patients [7]. In addition, the repetitive nature of ongoing training exercises may reduce patients’ motivation, thereby diminishing expected therapeutic benefits [8]. Fortunately, recent years have witnessed the emergence of virtual reality (VR) as a considerably promising technology for advancing motor function recovery—a potential stemming from its distinctive immersive and interactive features [9].

VR is defined as a computer-generated simulation of real-world environments that can be experienced by users [10], creating a realistic sense of immersion in which individuals experience a strong feeling of presence and contextual engagement in a three-dimensional space through multisensory input that engages vision, hearing, and touch [11]. VR-assisted rehabilitation can significantly improve upper-limb function in patients with stroke [7,12,13] as well as enhance balance, gait parameters, and postural control among patients with Parkinson’s disease and cerebral palsy [2,14]. The core strengths of VR lie not only in its effectiveness in increasing patient engagement in and adherence to exercise through contextualized and task-oriented training, but also in its capacity to provide immediate performance feedback, thereby effectively facilitating neuroplasticity and reinforcing motor learning processes [15]. Current evidence suggests that VR interventions can produce rehabilitation benefits comparable to those achieved with conventional therapy in several neurological populations and that combining VR with standard rehabilitation may reinforce functional outcomes [16].

With the development of emerging digital technologies, artificial intelligence (AI), machine learning, and deep learning have shown considerable potential in rehabilitation medicine, particularly in the field of motor rehabilitation [17]. These technologies can continuously track motor performance and dynamically adjust training tasks according to patients’ functional level, training status, and degree of engagement. In this way, they may improve the adaptability and personalization of rehabilitation interventions [18]. When artificial intelligence is combined with VR, AI-based personalized adjustment can not only enhance immersion and training motivation but also improve patients’ motor learning outcomes [19].

In addition, the wide application of sensor technology has opened new possibilities for VR in telerehabilitation [20]. By combining wearable sensors with motion capture systems, VR-based rehabilitation systems can quantitatively monitor patients’ movements [21] and support real-time feedback loops [22]. The addition of brain–computer interfaces (BCIs) and neurofeedback further extend this feedback mechanism. Head-mounted immersive VR combined with BCIs can translate patients’ motor intentions and brain activity into virtual movements with real-time feedback. This forms a closed-loop training system that enhances active participation and motor learning in neurorehabilitation [23].

VR is also increasingly combined with robot-assisted therapy and noninvasive brain stimulation. For example, a randomized controlled trial examined transcranial direct current stimulation (tDCS) combined with VR-based robot-assisted therapy in patients with upper-limb hemiplegia after subacute ischemic stroke. The combined intervention effectively improved upper-limb function and promoted activation of the ipsilesional primary motor cortex (M1) and the contralesional prefrontal cortex (PFC) [24]. Taken together, these developments suggest that VR is gradually shifting from a standalone interactive tool to an integrated digital rehabilitation platform that combines AI, sensors, gamification, robotics, and telerehabilitation.

Although the number of publications on VR for motor function rehabilitation has continued to grow and evidence regarding its intervention effects has become increasingly robust, studies that systematically map the overall development of this field remain relatively limited. Existing research has focused largely on evaluating the effectiveness of VR-based interventions, while comprehensive analyses of research structure, thematic evolution, and patterns of academic collaboration in the domain are still lacking. This gap makes it difficult for scholars to quickly grasp major research issues and future directions in this discipline, thus highlighting the need to adopt quantitative methods of systematically organizing and analyzing the developmental trajectory of VR-related rehabilitation research.

In this respect, bibliometric analysis, an approach based on bibliographic data [25], enables an objective depiction of the knowledge structure of a field from multiple perspectives, including publication trends, leading authors and institutions, national and regional distributions, keyword co-occurrences, co-citation networks, and emerging research frontiers [26]. These indicators are analyzed interactively through bibliometric methods, which thereby reveal shifts in research trends, identify representative research themes, and characterize the developmental stages of a field [27]. Accordingly, the present study carried out a systematic, bibliometric exploration of the literature on VR for motor function rehabilitation published from 2005 to 2025, with a view to clarifying dominant research topics and development trends, uncovering the knowledge structure of such scholarship and its evolutionary pathways, and providing a reference for future study and research agenda setting.

Therefore, this study aimed to answer the following research questions:

(1) What are the publication trends of VR research in motor rehabilitation from 2005 to 2025?
(2) What are the core journals in this field, and what disciplinary distribution do they represent?
(3) Which authors, institutions, and countries/regions are the major contributors to this field, and what collaboration patterns do they show?
(4) What are the main research hotspots and knowledge structures of this field based on keyword co-occurrence and thematic clustering analyses?
(5) What are the emerging frontier and future research trends of this field based on burst keyword analysis and timeline analysis?

## 2. Materials and Methods

### 2.1. Data Collection

The Web of Science Core Collection (WoSCC) is one of the most widely used scientific databases in the field of literature retrieval. As a bibliographic database, WoSCC provides standardized bibliographic and citation data, including citation counts, author affiliations, DOI information when available, keywords, and Keywords Plus, which makes it suitable for bibliometric analysis and knowledge mapping [28]. However, simply reporting “Web of Science Core Collection” as the data source is insufficient, as WoSCC consists of multiple sub-datasets with different coverage periods [29]. Therefore, this study specified the WoSCC editions used and their corresponding coverage periods to improve transparency and reproducibility. In the WoSCC search interface, all available editions were selected, including SCI-EXPANDED, SSCI, AHCI, CPCI-S, CPCI-SSH, and ESCI. The coverage periods differed across these editions: SCI-EXPANDED and SSCI cover records from 1900 to the present, AHCI from 1975 to the present, CPCI-S and CPCI-SSH from 1990 to the present, and ESCI from 2005 to the present. Although conference-related indexes were included in the initial search, conference papers and other non-eligible document types were excluded during screening. 

**Figure 1:**
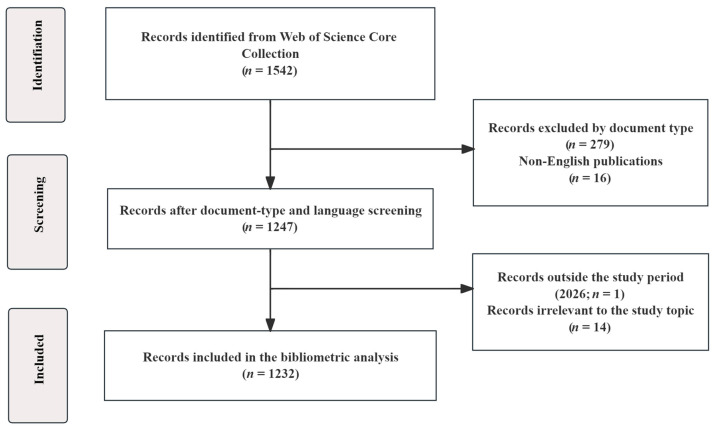
Screening process.

The inclusion criteria were as follows: (1) articles or reviews, (2) written in English, and (3) published from 1 January 2005 to 28 December 2025. Considering that relevant studies in this field before 2005 were limited and scattered, and that Holden’s 2005 article provided an early review of the application of VR in motor rehabilitation, this study selected 2005 as the starting year for the literature search [10]. The search strategy was as follows: TS = (“virtual reality*” OR “immersive virtual reality” OR “immersive VR”) AND TS = (“motor rehabilitation” OR “motor function rehabilitation” OR “motor recovery” OR “motor control” OR “motor learning” OR “functional motor”). This search strategy was adopted to ensure that the retrieved records specifically focused on virtual reality in motor rehabilitation. The final search was conducted on 28 December 2025. The initial search yielded 1542 records. After restricting the document types to articles and reviews and limiting the language to English, 1247 records remained. Subsequently, 15 records were removed, including one record published outside the study period in 2026 and 14 records that were irrelevant to the research topic. This left a final sample of 1232 records for bibliometric analysis. The screening process is detailed in Figure 1.

### 2.2. Data Analysis

Origin 2021, R software (version 4.5.2), VOSviewer (version 1.6.20), CiteSpace (version 6.4.R1), and Scimago Graphica (version 1.0.16) were used to perform the visualized bibliometric analyses. VOSviewer (version 1.6.20) was employed to generate and visualize country and institutional co-authorship networks, source co-citation networks, and keyword co-occurrence networks. Using Scimago Graphica (version 1.0.16), we created a geographical map of collaborations among different countries and regions.

CiteSpace 6.4.R1 was used to conduct keyword burst analysis and author collaboration network analysis. The time slicing was set from January 2005 to December 2025, with one year per slice. Link strength was calculated using the cosine similarity method, with the scope set to within slices. Node selection was based on the g-index, with the scaling factor set to *k* = 25. To simplify the network structure and retain the major connections, Pathfinder pruning and pruning of sliced networks were applied.

Keyword cleaning and standardization were conducted prior to all keyword-related analyses. A thesaurus file was used to merge singular and plural forms, spelling variants, abbreviations and their full forms, and synonymous expressions. For example, “VR,” “virtual reality (VR),” “virtual realities,” and “virtual environment” were merged into “virtual reality”; “poststroke” and “post stroke” were standardized as “post-stroke”; “upper limb,” “upper limbs,” “upper-limb,” “upper extremities,” “arm,” and “arms” were standardized as “upper extremity”; and “randomized clinical trial,” “randomized controlled trials,” and “rct” were merged into “randomized controlled trial.” In addition, an exclusion list was applied to remove generic or non-informative terms, such as “people,” “persons,” “programs,” “experience,” “system,” “impact,” and “technology.”

## 3. Results

### 3.1. Global Publication Trends

**Figure 2:**
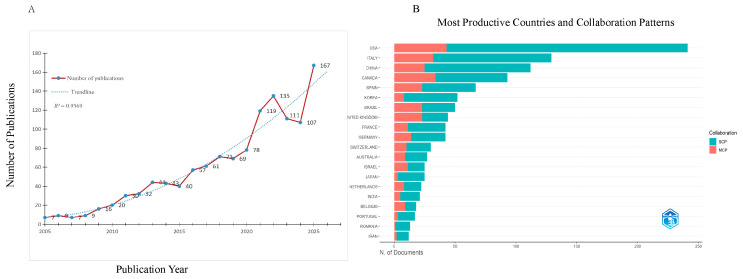
Trends in annual publication outputs (2005–2025) Note: (**A**) Yearly changes in publication output. (**B**) Distribution of corresponding authors by country and their collaboration patterns.

Figure 2A presents the annual publication trend of studies related to VR for motor rehabilitation. To further examine the overall trend, Microsoft Excel 2019 was used to generate a polynomial trendline using the Trendline function. The dotted line in Figure 2A represents the polynomial fitting curve created from the observed annual publication data. The trendline equation is *y* = 0.293*x*^2^ + 0.601*x* + 5.872, where *y* represents the number of publications and *x* represents the chronological sequence of publication years from 2005 to 2025. The model showed a good fit to the observed data (*R*^2^ = 0.9369), indicating a good fit of the trendline to the observed data, as an *R*^2^ value closer to 1 represents a stronger model fit. The fitted curve suggests a continued upward tendency in publication output in this field.

The annual publication pattern can be divided into three broad stages. From 2005 to 2014, the annual output was relatively small, suggesting the nascency of exploration. Beginning in 2015, publication output increased markedly, reaching 69 publications in 2019, which represents a 72.5% increase. From 2020 to 2025, the field entered a phase of rapid expansion, with the number of publications rising to 78 in 2020, increasing sharply to 119 in 2021, and reaching a peak of 135 in 2022. This increase may be partly related to the growing demand for home-based and telerehabilitation services during and after the COVID-19 pandemic, which may have encouraged further scholarly attention to the application of VR in motor rehabilitation [30]. The period 2023 to 2024 showed a slight decline in publication output, but it rebounded strongly in 2025 to 167 publications. Over the six-year period from 2020 to 2025, a total of 717 publications were produced, accounting for approximately 58.2% of all publications in the dataset. However, this publication growth should be interpreted with caution. In addition to the increasing scholarly interest in VR-based motor rehabilitation, the expansion of WoSCC coverage in recent years may have partially contributed to the observed increase in publication output.

### 3.2. Visualization of Country and Institutional Distribution

**Figure 3:**
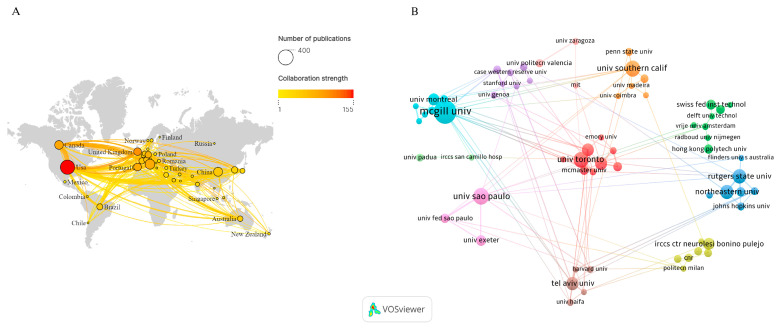
Global distribution and collaboration networks. Note: (**A**) Country collaboration map, circle size indicates the number of publications, and line color indicates collaboration strength. (**B**) Institutional collaboration network each circle represents an institution, and the circle size indicates the number of publications, different colors represent institutional collaboration clusters, and the lines indicate co-authorship links between institutions, the thickness of the lines reflects the strength of collaboration.

An analysis based on the corresponding authors’ countries/regions indicated that the leading contributor to publication was the United States (*n* = 241), followed by Italy (*n* = 129), China (*n* = 112), and Canada (*n* = 93). Figure 2B, along with the detailed information presented in Table 1, illustrates that the United States dominated the field, not only producing the largest number of publications but also maintaining close collaborative relationships with multiple countries. Although the United Kingdom registered a relatively lower total publication output, 52.3% of the studies conducted in the region involved multiple-country collaboration (MCP), pointing to a prominent pattern of international connectivity alongside national collaboration networks. Using Scimago Graphica (version 1.0.16), we further examined countries with more than five publications and generated a global visualization map of international collaboration (Figure 3A). In Figure 3A, the thickness of the connecting lines reflects the strength of collaboration between countries. The results showed that the United States, the United Kingdom, Canada, China, and Australia actively cooperated with other nations the world over. Meanwhile, scholars in East Asia and Europe exhibited strong interest in the application of VR to motor rehabilitation and, to some extent, established collaborative links with researchers in other countries.

At the institutional level, 1970 institutions were involved in research in the domain of interest. After the minimum publication threshold of five documents was set in VOSviewer (version 1.6.20), 126 institutions satisfied the inclusion criteria and were incorporated into the analysis of collaboration networks (Figure 3B). In terms of publication output, top ranking was achieved by McGill University (*n* = 34), followed by the University of São Paulo (*n* = 22), the University of Southern California (*n* = 21), the University of Toronto (*n* = 21), Rutgers State University (*n* = 19), and Northeastern University (*n* = 18). These findings indicate that the most productive institutions are concentrated in Canada and the United States, highlighting the clear strength of North America in this research area.

### 3.3. Visualization of Co-Cited Academic Journals

**Figure 4:**
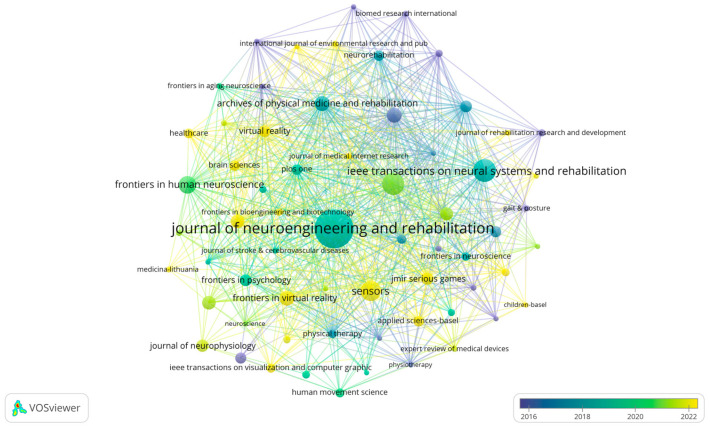
Co-cited journals related to VR in motor rehabilitation.

The journal co-citation analysis uncovered the overall intellectual structure characterizing the field in question as well as the characteristics of the journals operating in it [31]. These highly co-cited journals also reveal the interdisciplinary nature of VR-based motor rehabilitation. As can be seen in Table 2, the most frequently co-cited publication was the Journal of NeuroEngineering and Rehabilitation (*n* = 3350). Among the top 10 co-cited journals, more than half were ranked in the first quartile (Q1). In the network map of co-cited journals in Figure 4, the colors represent the average year in which the journals were cited. In recent years, journals such as JMIR Serious Games, Sensors, and Frontiers in Virtual Reality have been cited more frequently. This pattern may reflect a growing interest in technology-integrated rehabilitation research.

**Figure 5:**
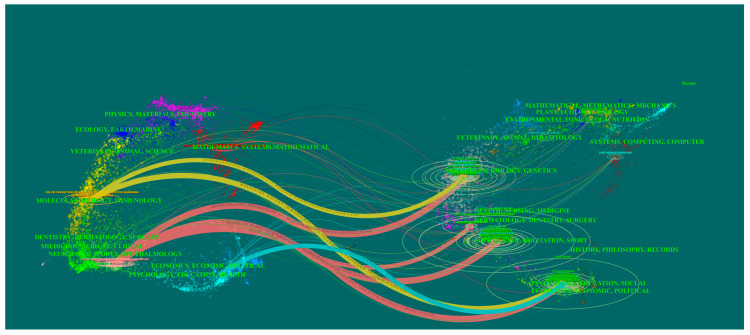
The dual-map overlay of journals on the application of VR in motor rehabilitation. The colored curves represent citation paths between citing journal disciplines on the left and cited journal disciplines on the right. Thicker curves indicate stronger citation relationships.

The journal dual-map overlay in Figure 5 illustrates the thematic distribution of academic journals. The left side represents the distribution of citing journals, while the right side represents the distribution of cited journals. The circles indicate the density and scale of journals in each thematic discipline, with large spheres suggesting a greater number of journals or higher citation volume in each field. The colored paths represent citation relationships between disciplines. A total of seven citation paths is shown in Figure 5. Notably, we observed a strong flow of citations from molecular biology, immunology, and genetics to neurology, sports science, ophthalmology, medicine, rehabilitation, and sports medicine. This pattern reflects that basic biomedical research provides a particularly strong knowledge base for neurorehabilitation and sports medicine.

### 3.4. Visualization of Collaboration Networks

**Figure 6:**
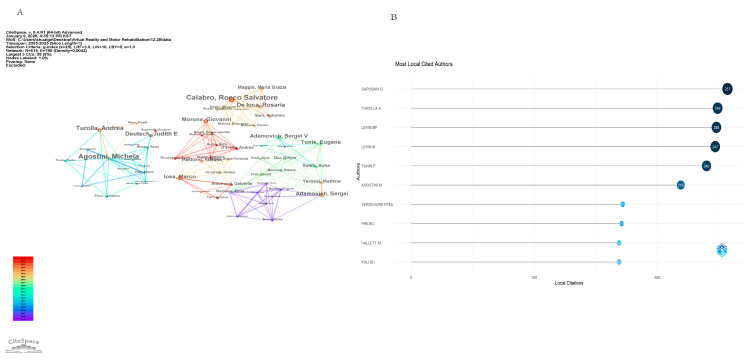
Author collaboration networks and local citation impact. Note: (**A**) Author co-authorship network. (**B**) Most locally cited authors.

As shown in Figure 6A, authors such as Agostini Michela and Calabrò Rocco Salvatore occupy relatively central positions with larger nodes and more connections, indicating extensive collaborative activity in this field. The network density was 0.0042, hinting at relatively dispersed overall collaboration among the authors. As presented in Table 3, Turolla A ranked first with 19 publications, followed by Levin MF (*n* = 18), Monteiro CBD (*n* = 15), Agostini M (*n* = 13), and Calabrò RS (*n* = 13). In terms of academic impact indicators, Levin MF had the highest h-index (14), indicating strong and relatively stable scholarly influence in the field. Turolla A and Monteiro CBD both had an h-index of 13, but the former achieved the highest g-index (19) among all the authors, implying that this author not only had substantial publication output but also wrote numerous heavily cited papers. The analysis of locally cited authors (Figure 6B) showed top ranking for Saposnik G with 257 local citations, followed by Turolla A (249), Levin MF (248), and Tonin P (240). Taken together, these findings suggest a certain degree of overlap between the most productive and the most influential authors.

### 3.5. Analysis of Co-Citation and Influential References

**Figure 7:**
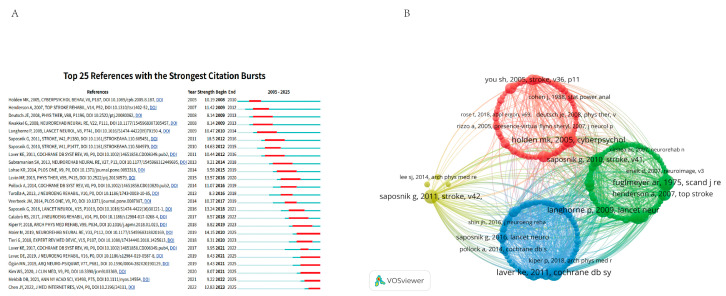
References with the strongest citation bursts and reference co-citation network. Note: (**A**) Top 25 references with the strongest citation bursts. (**B**) Reference co-citation network. The author–year labels shown in the figure correspond to the relevant numbered references cited in the References section [7,10,13,15,22,32,33,34,36,37,38,39,40,41,42,43,44,45,46,47,48,49,50,51].

To identify the research frontiers and knowledge base of VR for motor rehabilitation, we inquired into citation burst references and the reference co-citation network (Figure 7). Using CiteSpace 6.4.R1, we identified the 25 most influential references with the strongest citation bursts (Figure 7A). Two studies by Saposnik G—“Virtual reality in Stroke Rehabilitation: A Meta-Analysis and Implications for Clinicians” (strength = 18.50) and “Effectiveness of Virtual Reality Using Wii Gaming Technology in Stroke Rehabilitation: A Pilot Randomized Clinical Trial and Proof of Principle” (strength = 14.63)—showed prominent citation bursts for the periods 2012 to 2016 and 2012 to 2015, respectively. These findings indicate that VR interventions for stroke rehabilitation attracted substantial scholarly attention during these periods.

Judging from references that have continued to show citation bursts in recent years, the most prominent research has progressively focused on specific scenarios of VR application in stroke motor rehabilitation, particularly devoted to upper extremities. Relevant studies, such as “Effectiveness of Using Virtual Reality–Supported Exercise Therapy for Upper Extremity Motor Rehabilitation in Patients With Stroke: Systematic Review and Meta-analysis of Randomized Controlled Trials” (strength = 12.83) and “A Novel Fully Immersive Virtual Reality Environment for Upper Extremity Rehabilitation in Patients With Stroke” (strength = 9.22), have remained in a burst state, suggesting that the application of VR-based intervention to upper-limb functional recovery after stroke remains a particularly active and cutting-edge research direction.

The co-citation network pointed to the underlying knowledge structure of this field (Figure 7B), with several relatively distinct clusters of references formed in the network. Holden (2005) [10] was one of the early studies discussing the use of virtual environments in motor rehabilitation, providing an important conceptual basis for applying VR technology to therapeutic motor training. The two studies by Saposnik G (2010, 2011) [32,33] further strengthened the clinical evidence base by examining Wii-based VR intervention for stroke rehabilitation and synthesizing evidence on VR applications in stroke recovery. Langhorne P (2009) [34] provided a broader framework for understanding stroke rehabilitation and recovery, while Fugl-Meyer AR (1975) [35] introduced a standardized motor function assessment that has been widely used as an outcome measure in stroke rehabilitation research. Taken together, the central positions of these references in the co-citation network suggest that the knowledge base of VR-based motor rehabilitation is grounded not only in VR intervention studies, but also in broader stroke rehabilitation theory and standardized functional assessment.

### 3.6. Keyword Clusters and Thematic Evolution

**Figure 8:**
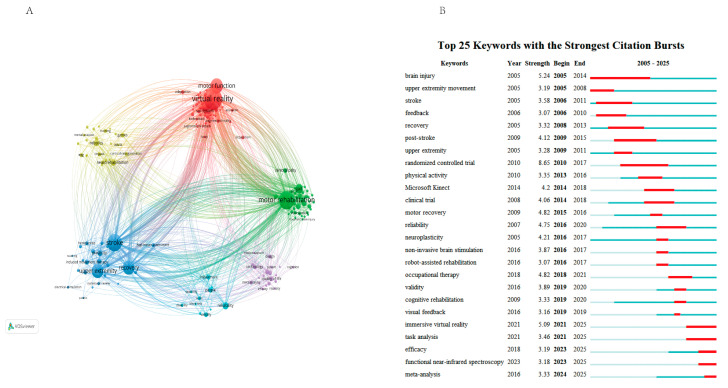
Keyword co-occurrence network and keyword burst analysis. Note: (**A**) Keyword co-occurrence network. (**B**) Top 25 keywords with the strongest citation bursts.

The examination of keyword clustering effectively identifies the critical areas of scholarship and developmental trends in an academic field. Using VOSviewer (version 1.6.20), we identified 188 keywords with a minimum occurrence threshold of 10 and constructed a keyword clustering map (Figure 8A). Five major thematic clusters were determined: Cluster 1 (red nodes in Figure 8A) was centered on “virtual reality” and included keywords such as “task system,” “biofeedback,” and “augmented reality,” reflecting that current research revolves principally around the design of VR-based rehabilitation tasks, the integration of feedback mechanisms, the application of augmented reality technologies, and related processes. Cluster 2 (green nodes) was centered on “motor rehabilitation” and “gait” and comprised keywords such as “cerebral palsy,” “intervention,” and “traumatic brain injury,” highlighting the application of VR in motor function interventions for the conditions. Cluster 3 (blue nodes) was concentrated in “stroke,” “recovery,” and “upper extremity,” indicating that research in this cluster focuses primarily on the role of VR in post-stroke functional recovery, particularly its clinical effects on upper-limb rehabilitation. Cluster 4 (yellow nodes) encompassed key terms such as “neurorehabilitation,” “robotics,” “EEG,” and “training,” reflecting the integration of VR with robotics and neuroscience-based approaches, including electroencephalographic monitoring. Cluster 5 (light blue and purple nodes) involved terms such as “quality of life,” “meta-analysis,” and measures of reliability and validity, pointing to growing attention to the evaluation of VR rehabilitation effectiveness, quality-of-life outcomes, and comprehensive evidence synthesis.

The keyword burst analysis is presented in Figure 8B. Five keywords continued to show bursts through 2025. Specifically, “immersive virtual reality” had the highest burst strength (strength = 5.09, 2021–2025), followed by “task analysis” (strength = 3.46, 2021–2025), “meta-analysis” (strength = 3.33, 2024–2025), “efficacy” (strength = 3.19, 2023–2025), and “functional near-infrared spectroscopy” (strength = 3.18, 2023–2025). Among these terms, “immersive virtual reality” showed both the highest burst strength and the longest burst duration, suggesting that immersive VR has received increasing attention in the included literature in recent years. The top 10 most frequent keywords and their corresponding betweenness centrality values are presented in Table 4. In addition, the CiteSpace 6.4.R1 keyword clustering network is provided in Supplementary Figure S1. The clustering analysis showed a modularity Q value of 0.5619 and a weighted mean silhouette value of 0.8357, with major clusters including VR-based motor rehabilitation, Parkinson’s disease, upper extremity, stroke rehabilitation, motor control, and immersive technology.

**Figure 9:**
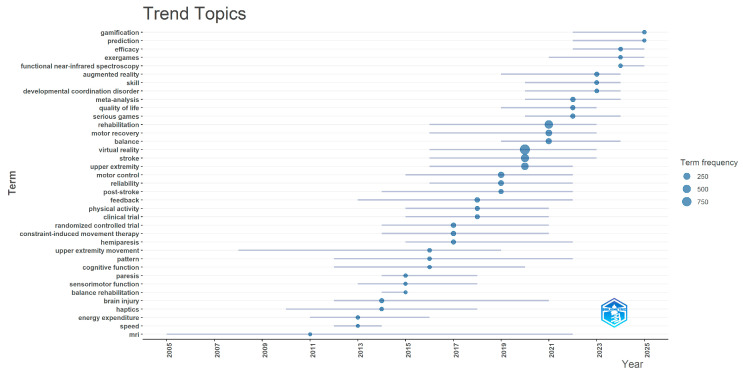
Temporal evolution of trends.

The bibliometrix package in R(version 4.5.2) was used to generate a thematic trend map of keywords (Figure 9) and visually represents the dynamic evolutionary trajectory of the field. As reflected in Figure 9, node size is proportional to keyword frequency, with larger nodes indicating the greater academic influence of a theme over the study period. The results demonstrated that the development of the domain followed a relatively clear temporal sequence.

In the earlier period before 2013, active terms included “mri,” “speed,” and “energy expenditure,” indicating that early studies were often associated with motor function measurement, sensory feedback, and neurological rehabilitation contexts. Between approximately 2013 and 2018, terms such as “feedback,” “upper extremity movement,” “randomized controlled trial,” and “constraint-induced movement therapy” became more visible, suggesting that the literature during this period increasingly involved intervention design, upper-limb rehabilitation, and clinical research methods. From 2019 onward, “virtual reality,” “stroke,” and “upper extremity” appeared with relatively large node sizes, reflecting their high frequency in the final dataset. More recent terms, including “gamification,” “prediction,” “efficacy,” and “functional near-infrared spectroscopy,” showed active timelines extending to 2025.

To further examine the maturity of major keyword themes, a thematic map was generated using the bibliometrix package in R (version 4.5.2) and is provided in Supplementary Figure S2. The thematic map showed that “rehabilitation–stroke–upper extremity” was positioned as a basic theme, indicating its central role in the field. “Virtual reality–gait–balance” showed relatively high relevance and moderate development, whereas “neuroplasticity–transcranial magnetic stimulation–brain–computer interface” appeared as a niche theme, suggesting a more specialized research direction.

## 4. Discussion

### 4.1. General Information

This study used bibliometric and visualization methods to analyze 1232 publications published between 2005 and 2025. Because bibliometric analysis is mainly used to map the structure, trends, and development of a research field, the findings of this study should be understood as descriptive and exploratory, with a focus on research patterns and potential future directions. The annual publication trend showed a gradual increase before 2020, followed by a marked rise after 2021. This pattern indicates growing scholarly attention to VR-based motor rehabilitation in recent years. The increase may be associated with several technological and contextual factors. Since the 2000s, VR research has expanded into health-related fields, including rehabilitation and neurosurgery [52]. Advances in head-mounted displays and software ecosystems have also created more favorable conditions for VR research [53]. Consumer-level devices such as HTC Vive, Oculus Rift, and Oculus Quest have made immersive VR more accessible and supported more interactive experiences through six degrees of freedom (6DoF) tracking [54]. In addition, the COVID-19 pandemic may have increased interest in digital health, telemedicine, and VR-supported rehabilitation [55]. Overall, the post-2020 increase in publications may reflect the combined influence of improved device accessibility, advances in motion-sensing technologies, and the broader expansion of digital rehabilitation research.

At the level of national publication output, the United States showed high productivity and active collaboration in research on VR-based motor rehabilitation. This may be partly related to its substantial investment in research and development and the availability of resources for interdisciplinary scientific work [31]. Because VR-based motor rehabilitation involves medicine, rehabilitation, human–computer interaction, computer science, and engineering, these research conditions may have supported the relatively high publication output of the United States within the WoSCC-indexed dataset analyzed in this study [56]. Meanwhile, Italy, China, Canada, the United Kingdom, and Australia showed relatively active collaborative relationships, suggesting broad international participation in this research field. These multinational collaboration patterns indicate active knowledge exchange across different healthcare and research contexts [57]. It should be noted that a higher number of publications does not necessarily indicate higher research quality, clinical relevance, or scientific impact. In terms of journal visibility and impact indicators, more than half of the top 10 co-cited journals were ranked in the Q1 quartile, suggesting that journals with high disciplinary visibility have played an important role in disseminating research in this domain.

Although the Journal of Neuro Engineering and Rehabilitation ranked first in co-citation frequency, its citation peak occurred around 2018. In recent years, JMIR Serious Games, Sensors, and Frontiers in Virtual Reality have been cited more frequently. Articles published in these journals are closely related to gamified rehabilitation, intelligent sensor-based monitoring [58], and immersive virtual interaction [59], respectively. These topics have become important sources of knowledge in VR-based motor rehabilitation in recent years.

Among the authors, Andrea Turolla from Italy produced the highest number of publications, indicating intensive research activity in VR-based motor rehabilitation. The author has also assembled a relatively stable research team with scholars such as Agostini Michela. Their work centers on stroke rehabilitation [42,60], postural stability in Parkinson’s disease [61], and the integration of VR with telemedicine [62]. Mindy F. Levin, from the Center for Interdisciplinary Research in Rehabilitation of Greater Montreal in Canada, achieved the highest h-index, suggesting both high productivity and sustained academic influence. The co-edited book, Virtual Reality for Physical and Motor Rehabilitation, further reflects Levin’s contribution to the synthesis of knowledge in this field [63].

Among locally cited authors, Gustavo Saposnik ranked first, which may be related to his involvement in the review “Virtual Reality for Stroke Rehabilitation” [13]. This review has been continuously updated and has been frequently referenced in studies on VR-based stroke rehabilitation. In addition, Calabrò Rocco Salvatore, Antonino Naro, and De Luca Rosaria are all affiliated with IRCCS Centro Neurolesi “Bonino Pulejo” in Italy, an institute with a strong focus on neurorehabilitation and emerging rehabilitation technologies. Their sustained productivity may reflect the institute’s long-term research focus on VR, robot-assisted rehabilitation, and telerehabilitation in motor recovery.

### 4.2. Dominant Research Issues and Trends

Post-stroke upper-limb functional recovery has long been one of the major research issues in the field of interest. Upper-limb motor impairment is common after stroke and can seriously affect the independence and quality of life of stroke survivors. Therefore, upper-limb rehabilitation remains an important need and challenge in stroke care [64]. A prospective controlled trial by Andrea Turolla et al. showed that VR training could significantly improve upper-limb motor function in patients with stroke [42]. Previous meta-analyses have reported positive effects of VR-supported motor therapy for the condition [7,13,65].

Nevertheless, the effects of VR interventions may vary according to rehabilitation stage, impairment severity, intervention design, and outcome measures. Current evidence suggests that VR-related interventions may produce relatively clear improvements in broader upper-limb outcomes, such as the Fugl-Meyer Assessment of Upper Extremity and the Functional Independence Measure [60,66]. These findings may help explain why upper-limb rehabilitation remained a central theme in the bibliometric results. However, outcomes related to hand dexterity, grasping ability, and fine motor control, such as the Box and Block Test and the Wolf Motor Function Test, have not shown similarly consistent improvements [66]. One possible explanation is that some VR systems may still have limited precision in capturing and providing feedback on subtle movements, particularly finger movements. Therefore, future VR rehabilitation systems may need to place greater emphasis on fine hand movement training, more precise motion detection, and individualized feedback.

Gait and balance rehabilitation for neurological disorders, particularly Parkinson’s disease and cerebral palsy, constituted another key research area. Previous studies have reported that VR-based rehabilitation may improve balance and gait outcomes in patients with Parkinson’s disease, and this finding is supported by meta-analytic evidence [67,68]. Studies on children with cerebral palsy have also suggested potential benefits of VR-based exergames for motor skills, arm function, postural control, and balance [3,14,69,70]. However, existing evidence remains limited by small sample sizes, heterogeneous intervention protocols, and differences in devices and outcome measures [14]. In addition, because many commercial VR gaming platforms are designed primarily for nondisabled users, VR systems and training tasks should be adapted to patients’ functional abilities [71]. Therefore, larger and more standardized clinical studies are needed to clarify the practical effects of VR-based gait and balance rehabilitation.

The extensive application of immersive VR has become one of the most prominent matters in recent years. The burst keyword analysis showed that “immersive virtual reality” has maintained a strong citation burst since 2021 (strength = 5.09) and continued through 2025, making it one of the burst terms with the longest durations and highest intensities. The latest explorations have increasingly focused on whether highly immersive intervention environments can improve motor rehabilitation outcomes after stroke [12,72,73].

Compared with conventional non-immersive systems, newer sensory-rich VR systems are often integrated with head-mounted displays, sensors, and motion-capture devices. These technologies may enhance presence, engagement, embodiment, and sensorimotor feedback during rehabilitation [74,75]. Some studies have also examined motivation, adherence, and neural responses in immersive VR-based rehabilitation [73,76,77]. Some recent studies have also used neuroimaging methods, such as functional near-infrared spectroscopy, to examine neural responses during immersive VR-based rehabilitation [78]. These studies reflect growing interest in more immersive, feedback-rich, and individualized rehabilitation approaches.

Although the discussion above accentuates the advantages of immersive VR on motor rehabilitation, its use is not entirely risk-free. Some patients have reported symptoms of VR sickness during use—effects that are generally associated with inconsistencies among visual, vestibular, and proprioceptive inputs [79]. In rehabilitation practice, however, a certain degree of sensorimotor mismatch may be intentionally introduced to challenge a patient’s motor system and advance motor learning and neuroplasticity. Even so, if such stimulation is carelessly designed, it may cause fatigue, frustration, and discomfort, which can reduce patients’ willingness to continue training [80]. Previous research suggests that VR sickness can be influenced by individual differences, the characteristics of head-mounted display, and the design of virtual content [81], but the effects of age and sex remain inconsistent across studies [82]. Therefore, the future development of immersive VR should transcend simple technological immersion and intervention effects. Greater attention should be given to comfort, safety, individual adaptability, and long-term acceptance during use.

Yet another crucial issue is the integration of VR and robot-assisted rehabilitation given the potential of robot-assisted therapy to pave the way for high-intensity, high-frequency, repetitive, and precise task practice, which is important for promoting neuroplasticity [83,84]. VR, meanwhile, can create task-specific, context-rich training environments that deliver multimodal and salient feedback, enhance immersion, and encourage patient engagement. It also enables meaningful repetitive practice, allows practice conditions to be manipulated, and integrates proprioceptive, visual, auditory, and vestibular processes to support motor skill acquisition and optimize motor learning [15]. Combining these two approaches may therefore satisfy the principal conditions conducive to neuroplasticity and enhance the clinical effects of rehabilitation training [85].

Combined robot-assisted and VR-based interventions may be equally beneficial for upper-limb function [86], balance control [87], kinematic performance, and health-related quality of life [88]. However, current evidence remains limited by considerable heterogeneity, differences in device standards, small sample sizes, inconsistent intervention protocols, and restricted outcome measures [85]. These challenges call for caution when interpreting findings related to robotics–VR combinations. Even so, the current research trend suggests a shift from simply testing the effectiveness of individual technologies to forays into integrated rehabilitation tasks and feedback mechanisms as well as more precise, interactive, and technology-integrated forms of intervention.

The COVID-19 pandemic may also have accelerated the development of VR-based telerehabilitation, which can help reduce barriers to rehabilitation access and increase patient motivation [89]. It possibly allows home-based patients to engage in longer and more frequent training. Compared with similar therapist-guided interventions delivered in clinical settings, telerehabilitation produces comparable improvements in functional outcomes [90]. On this basis, some scholars have proposed VR-based telerehabilitation as a complement to conventional rehabilitation [91]. Extending part of the training to the home environment may help augment training intensity and reduce time-related costs for both patients and healthcare institutions [92]. Nevertheless, the current evidence remains insufficient [91], as existing studies differ considerably with respect to intervention protocols, training intensity, device type, and outcome measures. Many feature small sample sizes and lack long-term follow-up [93]. Researchers are thus advised to include more robust randomized controlled trials to clarify the effectiveness of VR-based telerehabilitation, identify the populations most likely to benefit from it, and determine optimal implementation strategies.

Beyond robotics and telerehabilitation, emerging digital technologies may provide additional directions for VR-based motor rehabilitation. AI and machine learning may help interpret patients’ movement performance and adjust training tasks according to individual recovery status [17,18]. At the same time, wearable sensors and motion-capture systems can improve the monitoring of motor performance [94], especially for small-scale movements such as hand and finger actions that are often difficult to detect in conventional VR systems [95]. Brain–computer interfaces and neurofeedback may further extend VR-based rehabilitation by linking neural activity with motor tasks and supporting closed-loop training systems [96]. In addition, extended reality technologies, including augmented reality and mixed reality, may help connect virtual training tasks with real-world functional activities [97].

### 4.3. Strengths and Limitations

Based on publications indexed in the WoSCC, this study offered a broad overview of the research landscape, key research issues, and developmental trajectories of VR for motor rehabilitation. It aimed to help scholars better understand the current state of the field and identify promising avenues for future inquiry. That said, several limitations warrant consideration.

First, the analysis was confined to English-language publications indexed in the WoSCC, meaning that the literature reviewed here does not represent an exhaustive account of work produced in this area. Although WoSCC is widely used in bibliometric research because of its standardized citation records and suitability for citation-based analysis [98], its coverage is not evenly distributed across languages and disciplines. Previous studies have shown that WoS tends to overrepresent English-language journals and provides stronger coverage of natural sciences, engineering, and biomedical research than of social sciences and humanities [99]. In addition, recent evidence suggests that the expansion of WoSCC coverage may also influence publication growth patterns observed in bibliometric studies [100]. Some engineering-oriented studies, device-design papers, and technology-related conference proceedings may be more visible in engineering-focused or multidisciplinary databases, such as IEEE Xplore or Scopus, whereas clinically oriented rehabilitation studies may be more frequently indexed in PubMed or other rehabilitation-related databases. Therefore, some relevant clinical trials, rehabilitation practice studies, and technology-oriented conference papers not indexed in WoSCC may not have been captured in the present study. This may have influenced the observed publication trends, keyword structures, collaboration networks, and citation patterns. Future studies could integrate data from clinically oriented and rehabilitation-focused databases, or compare results across WoSCC, PubMed, and Scopus, to provide a more comprehensive and cross-validated understanding of this field.

A further limitation concerns the search strategy itself. While the search string was built around the core concepts of VR and motor rehabilitation, the keyword scope remained somewhat narrow, partly because the analysis relied on topic-based retrieval. Therefore, studies using related terms such as “XR,” “mixed reality,” “serious games,” or “exergaming” may not have been retrieved if they did not explicitly mention “virtual reality” or related VR terms. This may have influenced the keyword network and led to an underrepresentation of themes related to game-based rehabilitation, mixed reality, or engineering-oriented rehabilitation technologies. To address this limitation, future studies should consider broader search strategies, including terms related to specific disease types, intervention modalities, technological approaches, and rehabilitation goals. Bibliometric mapping by disease category, rehabilitation stage, and intervention context may also help clarify how VR is used across different rehabilitation settings. In addition, the technology-related topics identified in this study were derived from a VR-focused research corpus. Thus, they should be interpreted as themes related to VR-based motor rehabilitation, rather than as direct comparisons among distinct technology fields.

Another limitation concerns the retrieval date. Although the search was conducted on 28 December 2025, some articles published near the end of 2025 may not yet have been indexed in the WoSCC at the time of retrieval, which may have affected the completeness of the 2025 publication data. Future research could combine bibliometric analysis with systematic reviews, meta-analyses, or clinical studies to further examine how VR-based interventions are implemented and evaluated across different populations, rehabilitation stages, and outcome measures. Citation-based indicators may be influenced by publication year, because older papers have had more time to accumulate citations, while recently published studies may not yet have reached their full citation potential [101]. Therefore, future bibliometric studies could incorporate field- and time-normalized citation indicators to allow fairer comparisons of research impact across different publication years and research topics.

## 5. Conclusions

This study used visualization and bibliometric analysis tools to provide a bibliometric overview of WoSCC-indexed literature on VR technology for motor rehabilitation. The results showed that research in this area has grown rapidly overall, with a particularly marked increase observed after 2020. The United States, Italy, China, and Canada were the main contributors, and existing studies have concentrated on stroke, upper-limb functional recovery, gait and balance training, immersive VR, robot-assisted rehabilitation, telerehabilitation, and multimodal feedback. These findings suggest that the literature on VR-based motor rehabilitation has increasingly addressed clinical evaluation, technology integration, and application-oriented topics, moving beyond a narrow focus on early feasibility testing. These patterns also indicate growing interest in individualized training, immersive and feedback-rich rehabilitation environments, and multi-technology integration.

Nevertheless, several challenges remain. Intervention protocols are not yet sufficiently standardized, and evidence from clinical studies remains uneven across disease populations and outcome measures. Future clinical research, including high-quality randomized controlled trials and long-term follow-up studies, is needed to further evaluate the implementation and outcomes of different VR-based rehabilitation approaches. Technology-related topics such as immersive VR, robotics, and telerehabilitation were discussed as broad research themes in this study. Given the differences in their technical characteristics and rehabilitation contexts, future studies could conduct more fine-grained bibliometric analyses by technology type.

## Figures and Tables

**Table 1:** Top contributing countries/regions and collaboration patterns in articles on the application of virtual reality in motor rehabilitation.

Country	Articles	SCPs	MCPs	Frequency	MCP%
USA	241	198	43	926	17.8
Italy	129	97	32	438	24.8
China	112	87	25	409	22.3
Canada	93	59	34	365	36.6
Spain	67	44	23	226	34.3
Korea	52	44	8	153	15.4
Brazil	50	27	23	177	46
United Kingdom	44	21	23	165	52.3
France	42	31	11	184	26.2
Germany	42	28	14	161	33.3
Switzerland	30	20	10	142	33.3
Australia	27	18	9	113	33.3
Israel	25	14	11	106	44
Japan	25	22	3	92	12
The Netherlands	22	14	8	88	36.4
India	21	16	5	92	23.8

Note. SCPs: single-country publications; MCPs: multiple-country publications; Frequency: occurrence frequency of each country/region in the authors’ affiliation information.

**Table 2:** The top 10 journals with the most co-citations in the field of VR in motor rehabilitation.

Co-Cited Journal	Co-Citations	Impact Factor Based on Clarivate Analytics Journal Citation Report (2025)	JCR Quartile	Publisher	OA Status
Journal of NeuroEngineering and Rehabilitation	3350	5.2	Q1	BioMed Central/Springer Nature	Fully open access
Archives of Physical Medicine and Rehabilitation	1294	3.7	Q1	Elsevier	Hybrid open access
Neurorehabilitation and Neural Repair	1186	3.7	Q1	SAGE Publications	Hybrid open access
Frontiers in Human Neuroscience	1175	2.7	Q1	Frontiers Media	Hybrid open access
Frontiers in Neurology	876	2.8	Q2	Frontiers Media	Fully open access
IEEE Transactions on Neural Systems and Rehabilitation Engineering	863	5.2	Q1	IEEE	Fully open access
Frontiers in Neuroscience	854	3.2	Q2	Frontiers Media	Fully open access
Topics in Stroke Rehabilitation	851	2.5	Q1	Taylor & Francis	Hybrid open access
Sensors	666	3.5	Q2	MDPI	Fully open access
PLOS ONE	597	2.6	Q2	Public Library of Science	Fully open access

Note: Co-citations indicate journal co-citation frequency. Impact Factor and JCR Quartile were obtained from Clarivate Journal Citation Reports. Publisher refers to the journal’s publishing organization, and OA status indicates the journal’s open-access model.

**Table 3:** Top 10 most productive authors and their bibliometric impact in the field of VR in motor rehabilitation.

Author	Publications (n)	h_index	g_index	g_index	Centrality	Main Affiliation
Andrea Turolla	19	13	19	19	0	University of Bologna
Mindy F. Levin	18	14	18	18	0	McGill University
Carlos Bandeira de Mello Monteiro	15	13	15	15	0	University of São Paulo
Michela Agostini	13	10	13	13	0	IRCCS San Camillo Hospital
Rocco Salvatore Calabrò	13	9	13	13	0	IRCCS Centro Neurolesi “Bonino-Pulejo”
Talita Dias da Silva	12	12	12	12	0	University of São Paulo
Paweł Kiper	12	9	12	12	0	IRCCS San Camillo Hospital
Thais Massetti	11	10	11	11	0	University of São Paulo
Paolo Tonin	10	9	10	10	0	IRCCS San Camillo Hospital
Rosaria De Luca	9	9	9	9	0	IRCCS Centro Neurolesi “Bonino-Pulejo”

Note: Affiliations indicate the main or representative institutions identified from the authors’ publications in this field.

**Table 4:** Top 10 most frequent keywords and their betweenness centrality in the field of VR for motor rehabilitation.

Rank	Keyword	Frequency	Centrality
1	Virtual reality	886	0.06
2	Upper extremity	388	0.02
3	Rehabilitation	244	0.03
4	Stroke	164	0.04
5	Recovery	161	0.03
6	Gait	132	0.06
7	Motor learning	122	0.02
8	Balance	106	0.02
9	Motor rehabilitation	97	0.04
10	Reliability	96	0.06

## Data Availability

No new data were created or analyzed in this study. Data sharing is not applicable to this article.

## Supplementary Materials

The following supporting information can be downloaded at: https://www.mdpi.com/article/10.3390/healthcare14131976/s1, Supplementary Figure S1. Keyword clustering network generated by CiteSpace 6.4.R1; Supplementary Figure S2. Thematic map of major keyword themes based on centrality and density.

## Abbreviations

The following abbreviations are used in this manuscript:

WoSCC	Web of Science Core Collection
VR	Virtual Reality
AI	Artificial Intelligence
BCI	Brain–Computer Interface
EEG	Electroencephalography
tDCS	Transcranial Direct Current Stimulation
M1	Primary Motor Cortex
PFC	Prefrontal Cortex
XR	Extended Reality
AR	Augmented Reality
MR	Mixed Reality
fNIRS	Functional Near-Infrared Spectroscopy
6DoF	Six Degrees of Freedom
RCT	Randomized Controlled Trial
COVID-19	Coronavirus Disease 2019
TS	Topic
DOI	Digital Object Identifier
SCP	Single-Country Publications
MCP	Multiple-Country Publications
JCR	Journal Citation Reports
OA	Open Access
Q1	First Quartile
*R*^2^	Coefficient of Determination
SCI-EXPANDED	Science Citation Index Expanded
SSCI	Social Sciences Citation Index
AHCI	Arts & Humanities Citation Index
CPCI-S	Conference Proceedings Citation Index–Science
CPCI-SSH	Conference Proceedings Citation Index–Social Sciences & Humanities
ESCI	Emerging Sources Citation Index
