# Supplementary material for: Global Trends in Virtual Reality Research on Motor Rehabilitation from 2005 to 2025: A Bibliometric Analysis
Source: Healthcare (Basel). 2026 Jul 2;14(13):1976. doi: 10.3390/healthcare14131976 (PMC13360697; doi:10.3390/healthcare14131976)
Supplement: Supplementary file 1 [file healthcare-14-01976-s001.zip › healthcare-4346012-supplementary.pdf]

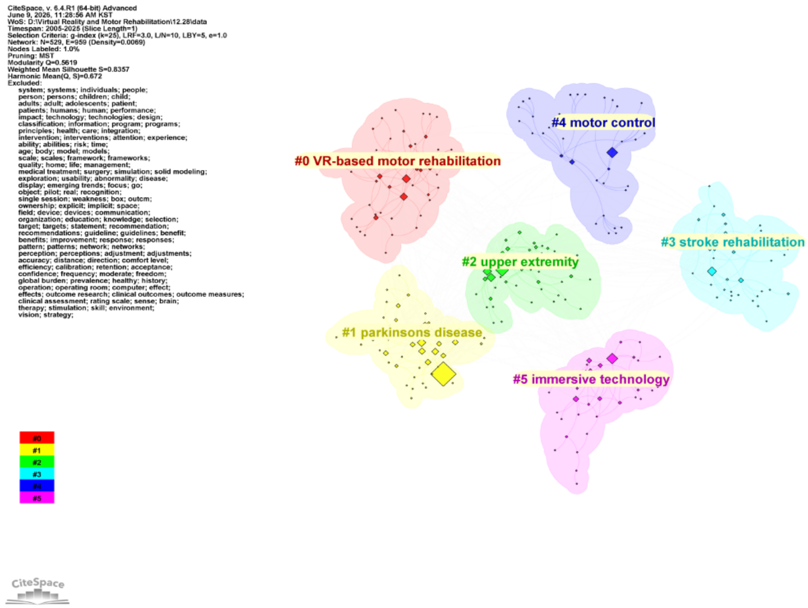

Supplementary Figure S1. Keyword clustering network generated by CiteSpace.

Note. The keyword clustering network was generated using CiteSpace 6.4.R1. The analysis covered the period from 2005 to 2025, with one year per slice. The clustering analysis yielded a modularity Q value of 0.5619 and a weighted mean silhouette value of 0.8357, indicating a clear modular structure and high internal consistency. The major clusters included VR-based motor rehabilitation, Parkinson’s disease, upper extremity, stroke rehabilitation, motor control, and immersive technology.

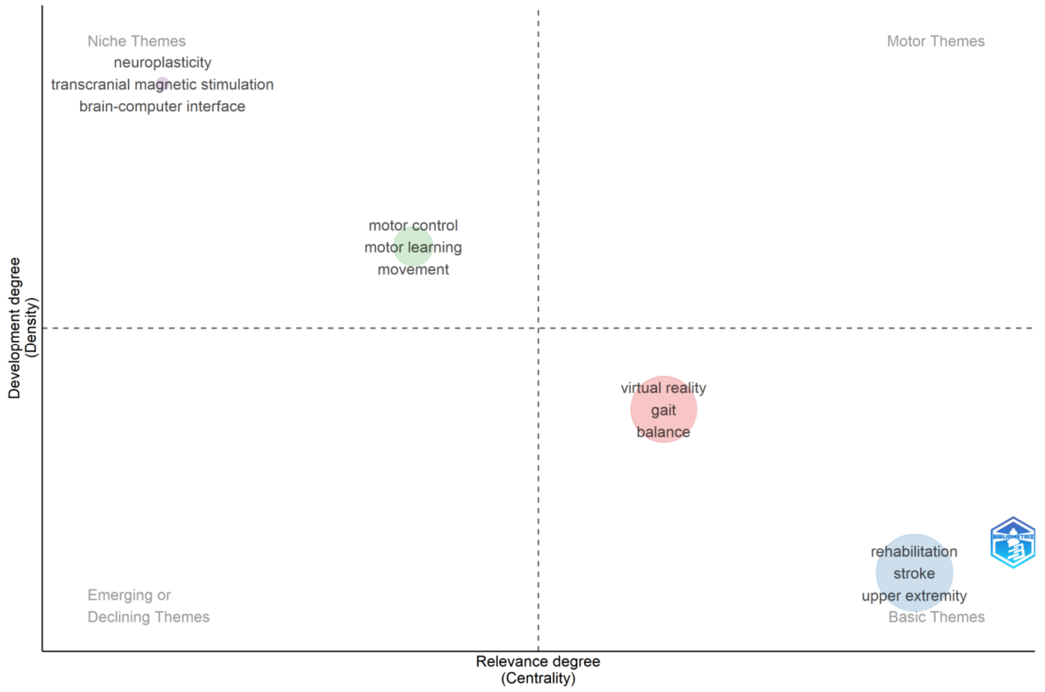

Supplementary Figure S2. Thematic map of major keyword themes based on centrality and density. The thematic map was generated using Biblioshiny. Centrality indicates the relevance of a theme to the overall research field, while density indicates the internal development degree of the theme.

The thematic map illustrates the relevance and development of major research themes in VR-based motor rehabilitation. The horizontal axis represents centrality, which reflects how strongly a theme is connected to the overall field. The vertical axis represents density, which indicates the internal development and maturity of a theme.

The themes “rehabilitation,” “stroke,” and “upper extremity” were located in the lower-right quadrant, representing basic themes. This suggests that these topics are closely connected to the field but are still developing internally. They can therefore be understood as foundational themes in VR-based motor rehabilitation, especially in relation to stroke rehabilitation and upper-limb recovery.

The theme including “virtual reality,” “gait,” and “balance” was also located on the right side of the map. This indicates that gait and balance training are highly relevant topics in this field. However, the relatively low density suggests that this theme has not yet become fully mature and may require further consolidation in terms of research focus and structure.

By contrast, “neuroplasticity,” “transcranial magnetic stimulation,” and “brain-computer interface” appeared in the upper-left quadrant, which represents niche themes. These topics showed relatively high internal development but lower centrality. This means that they are more specialized and less closely connected with the main body of VR-based motor rehabilitation research. They may reflect emerging directions related to neural mechanisms and neurotechnology-assisted rehabilitation.

The theme including “motor control,” “motor learning,” and “movement” was also positioned toward the upper-left area. This suggests that motor learning and movement control have developed as a related research topic, but they remain less central than stroke, rehabilitation, and upper-extremity recovery.

The thematic map indicates that VR-based motor rehabilitation is mainly organized around stroke, upper-extremity rehabilitation, gait, and balance. At the same time, more specialized topics, such as neuroplasticity, brain-computer interfaces, and transcranial magnetic stimulation, may represent future directions for mechanism-oriented and technology-integrated rehabilitation research.
